# Lifestyle Risk Factors of General and Abdominal Obesity in Students of the School of Medicine and Health Science of the University of Development Studies, Tamale, Ghana

**DOI:** 10.1155/2014/508382

**Published:** 2014-02-04

**Authors:** Victor Mogre, Rauf Nyaba, Samuel Aleyira

**Affiliations:** ^1^Department of Human Biology, School of Medicine and Health Sciences, University for Development Studies, P.O. Box TL 1883, Tamale, Ghana; ^2^Department of Allied Health Sciences, School of Medicine and Health Sciences, University for Development Studies, P.O. Box TL 1883, Tamale, Ghana

## Abstract

This study evaluated the prevalence of general and abdominal obesity among students of the University for Development Studies, School of Medicine and Health Sciences (UDS-SMHS), Tamale, Ghana. Also, lifestyle risk factors for the two obesity indices were investigated. This study was conducted among a sample of 646 students. Anthropometric measures of weight, height, and waist circumference were appropriately assessed. The prevalence of general and abdominal obesity was 1.9% and 4.2%, respectively. Risk factors of general obesity were being female (crude OR = 6.9, 95% CI = 1.85–25.80, *P* = 0.0021), engaging in light PA (OR = 12.45, 95% CI = 2.96–52.41, *P* = 0.0006), being aged 28–37 years (OR = 5.37, 95% CI = 1.39–20.68, *P* = 0.0329), nonintake of coffee (OR = 4.1, 95% CI = 1.10–15.28, *P* = 0.0357), being married (OR = 5.7, 95% CI = 1.48–22.02, *P* = 0.0286), and being abdominally obese (OR = 02.7, 95% CI = 25.61–11.60, *P* < 0.0001). Risk factors for abdominal obesity were being female, being married, having general obesity, and nonintake of coffee. Abdominal obesity was more prevalent than general obesity. Risk factors included being female, married, and generally obese and nonintake of coffee.

## 1. Introduction

Obesity has become a disease of public health concern for both developed and developing countries. Globally, obesity has been estimated to be the fifth leading cause of mortality [1]. The World Health Organization [2] estimates that 1.1 billion adults living in both developed and developing countries are overweight in which 300 million of them are obese. The prevalence of obesity in the USA has increased from 12.0% to 19.8% [3, 4] and half of its adult population are now overweight or obese [5]. An obesity prevalence of 10% has been reported in West Africa [6] and specifically 18% in the Republic of Benin [6]. In Ghana, a study conducted by Mogre et al. [7] in Tamale, Ghana, reported the prevalence of central obesity to be 31.2% among civil servants. Several studies have revealed that the prevalence of obesity in Ghana and other sub-Saharan African countries is increasing especially among women [7–9].

This growing epidemic is not only found in adults but also among children and young adults. The prevalence of obesity among school-aged children has more than tripled since the 1970s [10]. Several studies have demonstrated the childhood roots of adult obesity [11, 12]. Studies have reported a rising prevalence of childhood obesity in Ghana and other sub-Saharan countries [13–15]. However, there is limited data regarding obesity in the young adult group in Ghana. The few studies available used only BMI to assess the nutritional status of students studying various health related programs, a target group of particular interest as they are the future health care providers. The use of waist circumference (WC) in conjunction with BMI to assess abdominal and general obesity has not been extensively examined, particularly among Ghanaian students studying health related programs.

Both general and abdominal obesity are associated with increased risk of morbidity and mortality [16]. Abdominal obesity is a predisposing factor of cardiovascular diseases (CVDs), the main cause of obesity-related deaths. Traditionally, BMI has been chosen as an indicator to measure body size and composition and to diagnose underweight and overweight. Alternative measures such as WC, waist-to-hip ratio, and waist-to-height ratio that reflect abdominal adiposity have been suggested as being superior to BMI in predicting CVD risk. WC has been shown to be strongly predictive of all-cause mortality in young and middle-aged adults compared to older people and those with low BMI and could replace waist-to-hip ratio and BMI as a single risk factor for all-cause mortality [17].

The development of both general and abdominal obesity has been associated with potentially modifiable lifestyle factors such as physical inactivity, smoking, and alcohol consumption [18–20].

The aim of this cross-sectional study was to assess the prevalence of general and abdominal obesity among students studying health related programs in the University for Development Studies, School of Medicine and Health Sciences (UDS-SMHS), Tamale, Ghana. In addition, we investigated the effect of the lifestyle risk factors (age, gender, marital status, physical activity (PA), smoking, and alcohol and coffee consumption) on the two obesity indices (BMI and WC).

## 2. Methods

### 2.1. Participants

This cross-sectional study was executed between January and July 2013 among students studying health related academic programs in the University for Development Studies, School of Medicine and Health Sciences, (UDS-SMHS) Tamale, Ghana. From an eligible student population of 1809, a proportionate random sample of 646 (35.7% response rate) students that included more students from larger departments was selected to participate in the study with the help of a random number statistical table. Participants who were pregnant or had recent child births were excluded from the study. Demographic and sociocultural variables such as age, smoking status, and alcohol and coffee intake were also assessed. Participation in the study was voluntary and informed consent was obtained from each participant. The Ethics Committee of the University for Development Studies, School of Medicine and Health Sciences, Ghana, approved the study.

### 2.2. Anthropometric Variables

Anthropometric variables of waist circumference (WC), weight, and height were measured without shoes and with light clothing by trained personnel. WC was measured midway between the inferior angle of the ribs and the suprailiac crest [16]. During the measurement, participants stood in an upright position, with arms relaxed at the side, feet evenly spread apart, and body weight evenly distributed in accordance with the WHO expert consultation report on waist circumference and waist-hip ratio [16]. Abdominal obesity was determined as a waist circumference >102 cm in men and >88 cm in women according to the World Health Organization cutoff points and risk of metabolic complications for waist circumference [16]. Weight was measured to the nearest 0.1 kg using a UNICEF electronic scale manufactured by SECA. Prior to use, all scales were calibrated with a standard weight. Two measurements were taken and, if there were differences by a margin of more than 0.5 kg, a third measurement was taken and the mean of the closest two measurements was recorded. Height was measured to nearest 0.5 cm using a wall-mounted microtoise. Two measurements were made and compared and, if the measurements differed by more than 0.5 cm, a third measurement was taken and the mean of the closest two measurements was recorded. Body mass index (BMI) was calculated as BMI (kg/m^2^) = weight (kg)/[height (m^2^)]. General overweight and obesity were defined using the current World Health Organization definitions: underweight: BMI < 18.5 kg/m^2^, normal weight: BMI 18.5–24.9 kg/m^2^, overweight (preobese): BMI 25–29.9 kg/m^2^, and obese: BMI > 30 kg/m^2^ [21].

### 2.3. Physical Activity Level

The Global Physical Activity Questionnaire (GPAQ) was used to measure the level of physical activity of the participants [22]. The GPAQ consists of 16 questions about physical activity level in a typical week. The frequency and duration of time spent doing physical activity are measured in 3 domains: activity at work, travel to and from places, and recreational activities.

The GPAQ was used due to its standardization, easiness to administer, relative unobtrusiveness, and inexpensiveness. Its reliability and validity have been found to be 0.67–0.81 and 0.54, respectively [23]. Without modifications, the questionnaire was fully adapted for the study. However, to suit the Ghanaian context, local examples of types and intensity of activities were used. The GPAQ analysis protocol for the collection of all data and processing was followed [22]. All activity durations were converted into minutes. Energy expenditure, measured in metabolic equivalents (MET), was estimated using duration, intensity, and frequency of physical activities performed within 7 days. MET is the ratio of specific physical activity metabolic rates to the resting metabolic rate (1 MET = the energy cost of sitting quietly and was equivalent to a caloric consumption of 1 kcal/kg/hour). A MET-minute showed the total activity volume on a weekly basis and was calculated by multiplying time spent on each activity during a week by the MET values of each level of activity. Using the compendium of physical activities [24], MET values for various levels of activities were established. MET values of 4 and 8 were set for moderate-intensity (transport-related walking or cycling) and vigorous-intensity physical activity, respectively. Total MET/minutes/week was computed by the sum of all moderate- to vigorous-intensity physical activities performed at work, transport, and recreation. Based on the total MET/minutes/week, subjects were classified into light, moderate, and vigorous physical activity (PA) intensity as defined by the GPAQ analysis framework [22].

*Vigorous*. A participant is found within any of the following categories: vigorous-intensity activity on at least three days achieving at least 1,500 MET-minutes/weeks OR seven or more days of any combination of walking, moderate, or vigorous-intensity activities achieving at least 3,000 MET-minutes per week. 

*Moderate*. A participant is not achieving the criteria for the high category but either of the following three criteria: (a) 3 or more days of vigorous-intensity activities of at least 20 minutes per day OR (b) 5 or more days of moderate-intensity activities and/or walking of at least 30 minutes per day OR (c) 5 or more days of any combination of walking, moderate-, or vigorous-intensity activities accumulating at least 600 MET-minutes/week. 

*Light.* Participant's reported activity is lower than the categories outlined above or no activity is reported at all.

### 2.4. Statistical Analysis

The results were expressed as proportion and compared using Fischer's exact test or *χ* ^2^ for trend analysis as appropriate. A level of *P* < 0.05 was considered as statistically significant. GraphPad Prism version 5.00 (GraphPad Software, San Diego, California, USA, http://www.graphpad.com/) for Windows was used for statistical analysis.

## 3. Results

The general characteristics of the participants stratified by gender are recorded in Table 1. The mean age of the participating students was 23.06 ± 2.77 years. Significantly, male students (23.52 ± 2.83) were older than female students (22.03 ± 2.33). Only 1.1% of the students were smokers, 13.2% drank alcohol, and 57.1% drank coffee. More male (*n* = 29) students than female (*n* = 9) students were married. However, the differences were not significant when marital status of the students was stratified by gender using Fisher's exact test.

The mean BMI of the students was 21.79 ± 3.10 Kg/m^2^. The prevalence of general overweight and obesity was 9.3% and 1.9%, respectively. Whereas more male students (*n* = 404) than female students (*n* = 138) had normal weight, more female students than male students had general overweight (*n* = 42  versus  *n* = 18) and obesity (*n* = 9  versus  *n* = 3). The differences were significant when BMI status was stratified by gender using Fisher's exact test.

The prevalence of abdominal obesity was 4.2% (males: 0.7%, females: 11.9%, *P* < 0.0001).

In general, 14.7% engaged in light physical activity (PA) (males: 8.3%, females: 28.9%), 48.8% in moderate PA (males: 44.0%, females: 59.2%), and 36.5% in vigorous PA (males: 47.6%, females: 11.9%). The differences were significant when PA intensity was stratified by gender using Fisher's exact test.

BMI and WC statuses were stratified by age using Chi-square for trend analysis and presented in Table 2. The prevalence of general overweight and obesity was 10.0% and 1.8%, respectively, in students aged 18–24 years, 4.8% and 2.4% in those aged 25–31 years, and 30.0% and 0.0% in those aged 32–28 years. The differences were not significant when BMI status was stratified by age category using Chi-square for trend analysis.

The prevalence of abdominal obesity was 3.5% in students aged 18–24 years, 4.8% in those aged 25–31 years, and 0.0% in those aged 32–38 years.

**Figure 1:**
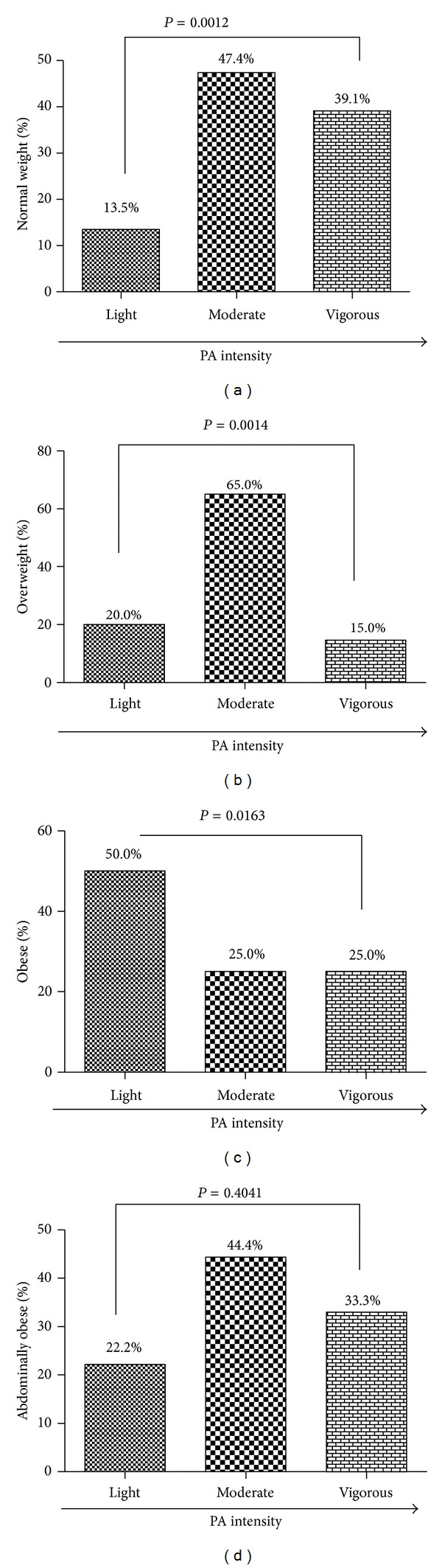
Association between PA intensity and normal weight (a), PA intensity and overweight (b), PA intensity and obesity (c), and PA intensity and abdominal obesity (d). Data was presented as proportion and analyzed using Chi-square for trend.

Using Chi-square for trend analysis, PA intensity was stratified by BMI and WC and presented in Figure 1. About 14.0% of normal weight students engaged in light PA, 47.4% in moderate PA, and 39.1% in vigorous PA. Generally, the prevalence of normal weight increased with an increase in PA intensity. Significantly, 85.0% of overweight students engaged in either light or moderate activity compared to 15.0% of them engaging in vigorous activity (*P* = 0.0014). The prevalence of obesity decreased significantly with an increase in PA intensity. Out of the 27 abdominally obese students, 22.2% participated in light PA, 44.4% in moderate PA, and 33.3% in vigorous PA. However, the differences were not significant when abdominal obesity was stratified by PA intensity using Chi-square for trend analysis.

The effect of different factors on the risk of developing obesity is recorded in Table 3. The factors that increased general obesity as determined by univariate analysis were being female, engaging in light PA, being aged 28–37 years, nonintake of coffee, being married, and being abdominally obese.

Table 4 shows the effect of lifestyle factors on the risk of developing abdominal obesity. Female students were severalfold at risk of developing abdominal obesity. Other lifestyle factors that increased the risk of developing abdominal obesity were nonintake of coffee, being married, and having general obesity.

## 4. Discussion

The current study brings to light the occurrence of general obesity and abdominal obesity and their associated risk factors coexisting with underweight among young adults in a developing country undergoing nutrition transition. It presents the problem of the double burden of malnutrition that requires a comprehensive approach to curb it. Results of this study emphasized the need for adopting a health policy to assess and control obesity and its risk factors among young adults in Ghana. University students provide an enormous opportunity for health authorities to design targeted programs to help curb the rising prevalence of obesity in older adults.

In the current study, the prevalence of general overweight and obesity was found to be 9.3% and 1.9%, respectively. Our findings concur with the 11.4% and 2.0% prevalence of overweight and obesity, respectively, among medical students in Delhi [25] and 10.4% prevalence of overweight among university students in Malaysia [26]. The prevalence of obesity in this study can be said to be among the lowest in literature in Sub-Saharan Africa and other countries. A study among university students living in halls of residence of the Nsukka campus of the University of Nigeria showed that 21.0% of the participants were obese [27]. Another study in 2008 among students of a Nigerian university found 23.9% and 3.4% of the study participants being overweight and obese, respectively [28]. In Malaysia, Gopalakrishnan et al. [29] found the prevalence of overweight and obesity to be 14.8% and 21.1% among medical students.

The prevalence of abdominal obesity measured by WC was found to be 4.2% and higher than the prevalence of general obesity. This corresponds with the findings of Hazizi et al. [26] among university students in which the prevalence of abdominal obesity was found to be 17.2% as determined by WC and 3.4% as determined by BMI. WC is more sensitive than BMI. Waist circumference correlates more closely with abdominal adipose tissue than BMI [16]. It is much easier and more accurate to measure WC than to measure weight and height [16]. In addition WC is a strong predictor of all-cause mortality in young and middle-aged adults compared to older people and those with low BMI [17]. Measures of abdominal obesity have also been shown to be better than BMI as predictors of CVD risk, although combining BMI with these measures may improve their discriminatory capability [30]. This suggests that relying on only BMI might underestimate the proportion of obese students and those at risk of developing CVDs and diabetes.

Generally, studies in older Ghanaians had a higher prevalence of obesity than seen in this study [19, 31, 32]. The implication of this finding is that efforts at addressing general and abdominal obesity in the adult population should commence at younger ages. This will help to address chronic diseases such as CVDs and diabetes. University students could be appropriate targets for such interventions.

The prevalence of both general and abdominal obesity was significantly higher in female than in male students. Using univariate analysis female students were severalfold at risk of developing general and abdominal obesity. This concurs with several studies conducted among young and older adults in Sub-Saharan Africa [19, 27, 28, 31, 33, 34]. This could be due to the fact that more females than males significantly participated in light PA. Findings of the current study showed that participants who engaged in light PA were severalfold at risk of becoming obese as determined by BMI. The higher prevalence of general and abdominal obesity in female students could also be as a result of the notion that being obese is considered as a sign of well-being in Africa. A study among university students in Nigeria showed that most subjects believed that being obese gives respect and is a sign of living well [35].

Interestingly, nondrinkers of coffee were at 4.1 and 4.2 times at risk of developing general and abdominal obesity respectively. This presents an inverse relationship between coffee consumption and body fat. Findings on the effect of coffee on body fat have been inconsistent. A cross-sectional study among a noninstitutionalized US population showed that coffee consumption was not related to BMI or WC in either gender [36]. However, a study by Lopez-Garcia et al. [37] found that an increase in coffee consumption was associated with less weight gain. Caffeine, the chemical found in coffee, has been shown to have an effect on weight gain and weight loss. Caffeine has been shown to stimulate the utilization of fat in muscle tissue during exercise [38]. In addition, Astrup et al. [39] reported a dose-dependent increase in basal energy expenditure with caffeine intake in healthy subjects who had moderate habitual caffeine consumption attributing the effect to an increase in lactate and triacylglycerol production and increased vascular smooth muscle tone. Acheson et al. [40] suggested that caffeine may stimulate thermogenesis by increasing lipid turnover. All the above mechanisms suggest a beneficial effect of caffeine on energy metabolism thereby resulting in weight loss. A limitation of our study was that information on coffee consumption was self-reported. In addition the quantity, flavor, and frequency of coffee consumed were not also assessed.

Students who were married were 5.7 and 6.1 times at risk of developing general and abdominal obesity. In consonance with our findings a nationwide cross-sectional survey in Iran found that currently and formerly married individuals were more overweight or obese than those never married [41]. Several cross-sectional studies have reported similar relationships [42–45]. However, other studies have also reported inconsistent findings ranging from the lack of association [46, 47] to a protective effect of marriage [48]. The positive relationship between marital status and obesity or abdominal obesity found in this study could be related to the fact that married people engage in less physical activity after marriage, have a more stable dietary pattern, and may place less emphasis on being attractive [41].

Using Chi-square for trend analysis age was not associated with obesity or with abdominal obesity. However, using univariate analysis participants aged 28–37 were 5.7 times at risk of developing obesity. This is in keeping to the fact that general and abdominal obesity increase with increase in age. This is in agreement with several studies conducted among young [49] and older adults [50–52]. The proportion of fat deposited in the abdomen increases as body shape becomes more android with age, due to decreasing height and increasing slackness of abdominal wall muscles. During adulthood, weight gain occurs in the abdominal region, emphasizing the importance of hypertrophic obesity, which is generally android [53]. This change in the adult figure may influence the positive association between age and excess abdominal adiposity [54].

Students who were abdominally obese were severalfold at risk of developing obesity and those generally obese were also severalfold at risk of developing abdominal obesity. A longitudinal, population-based birth cohort study conducted by Laitinen et al. [55] among 31-year-old men found high BMI as an important predictor of abdominal obesity.

Even though smoking has been shown to be associated with general and abdominal obesity in some studies [56, 57], a significant association was not found in this study. This could be due to the fact that a small proportion of the sample ever smoked. Notwithstanding, several other studies have also found no significant association between smoking and obesity [55].

Alcohol consumption was not a significant risk factor for both obesity and abdominal obesity. Several conflicting findings have been reported on the effect of alcohol on obesity ([58–61]). A study among randomly selected adults aged ≥18 years in Brazil showed that participants who consumed alcohol had a higher probability of being centrally obese among men and women [62]. A similar association has also been reported among Korean adults [63]. Other studies report that light alcohol drinking is known to be associated with lower incidence of obesity [56]. Another study among middle-aged women found that the risk of becoming overweight was almost 30 percent lower for women who consumed one or two alcoholic beverages a day, compared to nondrinkers [64]. The lack of association in our study could be due to the fact that quantity and frequency of alcohol consumed were not measured and students self-reported their consumption status.

Our study is not without limitations. This study was conducted on a sample of only university students without considering young adults from other nonuniversity tertiary students and those out of school. This makes it difficult to generalize the findings to the entire young adult population in Ghana. Even though the findings of this study are comparable to previous studies, its cross-sectional design makes it unable to establish causality.

## 5. Conclusion

The prevalence of abdominal obesity was higher than general obesity. Associated risk factors of both general and abdominal obesity were being female, engaging in light PA, being married, and nonintake of coffee. Health authorities should design health and nutrition education programs that target young adults in our tertiary institutions.

## Figures and Tables

**Table 1:** General characteristics of the participants (*n* = 646).

Variable	Total (*n* = 646)	Male (*n* = 445)	Female (*n* = 201)	*P* value
Mean ± SD (age)	23.06 ± 2.77	23.52 ± 2.83	22.03 ± 2.33	<0.0001
Smoking				
Yes	7 (1.1%)	7 (1.3%)	0 (0.0%)	0.1055
No	639 (98.9%)	438 (98.4%)	201 (100.0%)	
Drinks alcohol				
Yes	85 (13.2%)	70 (15.7%)	15 (7.5%)	0.8805
No	561 (86.8%)	375 (84.3%)	186 (92.5%)	
Drinks coffee				
Yes	369 (57.1%)	260 (58.4%)	109 (54.2%)	0.3451
No	277 (42.9%)	185 (41.6%)	92 (45.8%)	
Married				
Yes	38 (5.9%)	29 (6.5%)	9 (4.2%)	0.3689
No	608 (94.1%)	416 (93.4%)	192 (90.6%)	
BMI status				
Underweight	32 (5.0%)	20 (4.5%)	12 (6.0%)	0.4367
Normal weight	542 (83.9%)	404 (90.8%)	138 (68.7%)	<0.0001
Overweight	60 (9.3%)	18 (4.0%)	42 (20.9%)	<0.0001
Obesity	12 (1.9%)	3 (0.7%)	9 (4.5%)	0.0021
Mean ± SD (BMI)	**21.79 ± 3.10**	**21.32 ± 2.33**	**22.84 ± 4.15**	<0.0001
Abdominal obesity			
Yes	27 (4.2%)	3 (0.7%)	24 (11.9%)	<0.0001
No	619 (95.8%)	442 (99.3%)	177 (88.1%)	
Mean ± SD	**77.36 ± 6.45**	**76.32 ± 4.79**	**79.65 ± 8.74**	<0.0001
Physical activity				
Light	95 (14.7%)	37 (8.3%)	58 (28.9%)	<0.0001
Moderate	315 (48.8%)	196 (44.0%)	119 (59.2%)	0.0005
Vigorous	236 (36.5%)	212 (47.6%)	24 (11.9%)	<0.0001

Data are presented as proportion and compared using Fischer's exact test.

**Table 2:** BMI status and abdominal obesity stratified by age.

Variable	18–24 (*n* = 510)	25–31 (*n* = 126)	32–38 (*n* = 10)	Chi-square	*P* value
BMI status					
Underweight	28 (5.5%)	4 (3.2%)	0 (0.0%)	1.67	0.1965
Normal weight	422 (82.7%)	113 (89.7%)	7 (70.0%)	1.13	0.2879
Overweight	51 (10.0%)	6 (4.8%)	3 (30.0%)	0.22	0.6411
Obesity	9 (1.8%)	3 (2.4%)	0 (0.0%)	0.03	0.8533
Abdominal obesity					
Yes	18 (3.5%)	6 (4.8%)	0 (0.0%)	0.07	0.7918
No	492 (96.5%)	120 (95.2%)	10 (100.0%)	—	—

Data was presented as proportion and analyzed using Chi-square for trend.

**Table 3:** Risk factors for general obesity using univariate analysis.

Variable	*n*/N*	Rate of obesity	OR (95% CI)	*P* value
Gender				
Male	3/445	0.7%	6.9 (1.85–25.80)	0.0021
Female	9/201	4.5%	Ref	Ref
Physical activity				
Light	6/37	16.2%	Ref	Ref
Moderate	3/196	1.5%	12.45 (2.96–52.41)	0.0006
Vigorous	3/212	1.4%	13.48 (3.21–56.72)	0.0004
Age				
18–27	9/605	1.5%	5.4 (1.39–20.68)	0.0329
28–37	3/41	7.3%	Ref	Ref
Smoking				
Yes	0/7	0.0%	Ref	Ref
No	12/639	1.9%	3.3 (0.18–61.91)	1
Alcohol consumption				
Yes	0/85	0.0%	Ref	Ref
No	12/561	2.1%	0.3 (0.02–4.39)	0.3825
Drinks coffee				
Yes	3/369	0.8%	4.1 (1.10–15.28)	0.0357
No	9/277	3.2%	Ref	Ref
Marital status				
Yes	3/38	7.9%	Ref	Ref
No	9/608	1.5%	5.7 (1.48–22.02)	0.0286
Abdominal obesity				
Yes	9/27	33.3%	Ref	Ref
No	3/619	0.5%	102.7 (25.61–11.60)	<0.0001

*Number of subjects with general obesity/number of subjects in each category. Ref refers to reference point.

**Table 4:** Risk factors for abdominal obesity using univariate analysis.

Variable	*n*/N*	Rate of abdominal obesity	OR (95% CI)	*P* value
Gender				
Male	0/445	0.0%	123.0 (7.43–2035.00)	<0.0001
Female	24/201	11.9%	Ref	Ref
Physical activity				
Light	6/95	6.3%	Ref	Ref
Moderate	12/315	3.8%	1.7 (0.62–4.67)	0.3888
Vigorous	6/236	2.5%	2.6 (0.81–8.23)	0.1102
Smoking				
Yes	0/7	0.0%	0.6 (0.03–10.76)	1.0000
No	24/639	3.8%	Ref	Ref
Drinks alcohol				
Yes	3/85	3.5%	1.1 (0.310–3.64)	1.0000
No	21/561	3.7%	Ref	
Drinks coffee				
Yes	6/369	1.6%	4.2 (1.65–10.74)	0.0014
No	18/277	6.5%	Ref	Ref
Age				
18–27	21/605	3.5%	2.2 (0.63–7.69)	0.1902
28–37	3/41	7.3%		
Marital status				
Yes	6/38	15.8%	Ref	Ref
No	18/608	3.0%	6.1 (2.28–16.54)	0.0017
Obesity				
Yes	9/12	75.00%	Ref	Ref
No	15/634	2.40%	123.8 (30.41–503.90)	<0.0001

*Number of subjects with distortion/number of subjects in each category. Ref refers to reference point.
